# Age-Related White Matter Changes

**DOI:** 10.4061/2011/617927

**Published:** 2011-08-23

**Authors:** Yun Yun Xiong, Vincent Mok

**Affiliations:** Department of Medicine and Therapeutics, The Chinese University of Hong Kong, Hong Kong Special Administrative Region, Shatin 999077, Hong Kong

## Abstract

Age-related white matter changes (WMC) are considered manifestation of arteriolosclerotic small vessel disease and are related to age and vascular risk factors. Most recent studies have shown that WMC are associated with a host of poor outcomes, including cognitive impairment, dementia, urinary incontinence, gait disturbances, depression, and increased risk of stroke and death. Although the clinical relevance of WMC has been extensively studied, to date, only very few clinical trials have evaluated potential symptomatic or preventive treatments for WMC. In this paper, we reviewed the current understanding in the pathophysiology, epidemiology, clinical importance, chemical biomarkers, and treatments of age-related WMC.

## 1. Introduction

Age-related white matter changes (WMC) are prevalent findings among the elderly.WMC are considered to be etiologically related to cerebral small vessel disease and are important substrates for cognitive impairment and functional loss in the elderly [1].Although extensive studies have investigated various aspects on WMC, controversies still exist in the pathophysiology and clinical phenotypes, and consensus regarding to treatments for WMC has not been reached.In this paper, we aimed to provide an update review on the epidemiology, pathophysiology, neuroimaging, clinical importance, chemical biomarkers, and treatments of age-related WMC. 

The literature search was conducted using the National Center for Biotechnology Information (NCBI) PubMed/Medline to identify relevant articles related to WMC that were published until June 2011. We used the following keywords for the search: white matter, white matter changes, white matter lesions, leukoaraiosis, white matter hyperintensities, and small vessel disease. 

The articles were included in this paper if (1) the journal article was published in English and (2) they were related to epidemiology, pathophysiology, neuroimaging, genetics, clinical phenotypes, biomarkers, and treatment of WMC. Further searches on bibliographies in the main articles and relevant papers were performed.

## 2. Prevalence and Risk Factors

WMC are almost endemic in community elderly with prevalence ranging from 50% to 98% [2–6]. In stroke patients, prevalence of WMC varies from 67% to 98% [7–10]. In Alzheimer's disease, WMC are also common with prevalence ranges from 28.9% to 100% [11–13]. About 30–55% of patients with Parkinson's disease (PD) also harbor WMC [14–16]. Age [2, 4, 6, 17–20] and hypertension [3, 18, 20–30] are established risk factors for WMC. A recent Manhattan study in community elderly found that compared with individuals with low blood pressure (BP) and low fluctuations in BP, the risk of WMC increased with higher BP and BP fluctuations [31]. Associations of diabetes mellitus (DM), cholesterol, smoking, and homocysteine are less consistent between studies. Although past studies had suggested that WMC are highly heritable [32] and that several polymorphisms in various candidate genes, such as apolipoprotein E (epsilon 4±), methylenetetrahydrofolate reductase (677 cytosine/thymine polymorphism (C/T)), and angiotensinogen (Met235Thr), were found to be associated with WMC, [33–35] a recent meta-analyses failed to show convincing evidence for an association between WMC and the candidate genetic polymorphisms [36].

## 3. Progression of WMC

Age-related WMC are not static lesions. The lesions may progress, or even regress, over time. Several longitudinal studies have investigated the rate and predictors for progression of WMC [37–47]. Perhaps the most consistent predictor for progression of WMC is the baseline severity of WMC [44, 47, 48]. Patients with punctate WMC usually have minimal progression of WMC, whereas those with early confluent and confluent WMC at baseline have rapid progression of WMC [44, 49]. In the Austrian Stroke Prevention Study, the median (interquartile range) volume increase over the 6-year period was 0 cm^3^ in subjects with no lesions, 0.2 (0.0–1.1) cm^3^ in subjects with punctuate lesions, 2.7 (0.5–5.9) cm^3^ in subjects with early confluent lesions, and 9.3 (7.1–21.0) cm^3^ for individuals with confluent WMC at baseline [44]. In AD and PD patients, the baseline severity of WMC also predicted lesion progression, AD median WMC progression was 0.08%, while PD dementia was 0.07% [50]. Sachdev et al. study in 51 healthy subjects with follow-up duration of 6 years found that increase in DWMC volume (43.8%) was greater than that of PVWMC (29.7%) [47]. Furthermore, female may have more lesion progression than male. A longitudinal study in 554 elders (313 men, 241 women) aged 70 to 82 years indicated that women had significantly higher DWMC volume than men at baseline; after 3 years followup, they had accumulated approximately twice as much DWMC as men, whereas their progression of PVWMC was similar to men [51]. Other factors associated with faster decline in WMC are higher age, cigarette smoking, and elevated BP [48].

## 4. Pathology and Physiology

Pathologically, WMC are characterized by partial loss of myelin, axons, and oligodendroglial cells; mild reactive astrocytic gliosis; sparsely distributed macrophages as well as stenosis resulting from hyaline fibrosis of arterioles and smaller vessels [52]. Nowadays, the most accepted opinion is that WMC represents incomplete ischemia mainly related to cerebral small vessel arteriolosclerosis [53]. 

Another mechanism is blood-brain barrier dysfunction. Small vessel alterations could lead to damage of the blood-brain barrier and chronic leakage of fluid and macromolecules in the white matter [53]. Increased concentration of cerebrospinal fluid albumin and IgG values were found in patients with CT-detected WMC [54, 55]. A recent MRI study even found that blood-brain barrier permeability increased in normal-appearing white matter in patients with WMC and its presence in normal-appearing white matter would be consistent with it playing a causal role in disease pathophysiology [56]. Moreover, a pathological study showed that albumin extravasation was widespread in the ageing brain and enhanced in WMC [57]. According to the location of the lesions, WMC can be divided into periventricular WMC (PVWMC) and deep WMC (DWMC). Pathological studies have shown that PVWMC were related to disruption of the ependymal lining with subependymal widening of the extracellular space resulting from disruption of the blood brain barrier, whereas the DWMC were mainly related to incomplete ischemic arteriolosclerosis [58, 59].

Vascular risk factors, especially hypertension, cause lipohyalinosis of the media and thickening of the vessel walls, which attributes to narrowing of the lumen of the small perforating arteries and arterioles nourishing the deep white matter [60]. The perforating vessels, which originate from cortical and leptomeningeal arteries, have a relatively poor anastomotic system, which makes the white matter vulnerable to cerebral ischemia. Hypertension can also cause disturbances in the blood-brain barrier and lead to WMC by cerebral edema, activation of astrocytes, or destructive enzymes or other poisons which pass through the damaged vessel walls [60]. DM alters the glucose and insulin transfer across the blood-brain barrier, thus affects regional metabolism and microcirculation. Chronic hyperglycemia, which further alters membrane permeability and decreases regional blood flow, might lead to permanent cell damage. Therefore, DM seems to be associated with progressive metabolic disturbance in the cerebrovascular bed that may affect blood flow and accelerate the white matter ischemia [61, 62].

Recently, postmortem Medical Research Council Cognitive Function and Ageing Study using RNA microarray and pathway analysis found that 8 major pathways in which multiple genes showed altered RNA transcription (immune regulation, cell cycle, apoptosis, proteolysis, ion transport, cell structure, electron transport, metabolism) and WMC represented areas with a complex molecular phenotype [63]. Xu et al. study revealed that 241 genes specific for WMC expression were associated with inflammation, oxidative stress, detoxification, and hormonal responses, included genes associated with brain repair, long-term potentiation, and axon guidance, and included genes associated with oligodendrocyte proliferation, axon repair, long-term potentiation, and neurotransmission [64]. These neurogenetic findings support the ischemia, blood-brain barrier dysfunction, systemic oxidative stress, and inflammation in the pathogenesis of WMC, as well as other potential processes in the pathogenesis which warrant future research. 

Other mechanisms hypothesized to be involved in the pathophysiology of WMC encompassing dysfunction of vasomotor reactivity and autoregulation [65–69], chronic edema [70, 71], apoptosis [72], and endothelial dysfunction [73, 74].

Therefore, the pathophysiology of WMC is complex and may be multifactorial. Further studies should address more on how these different pathways interact with each other.

## 5. Neuroimaging Assessment

**Figure 1:**
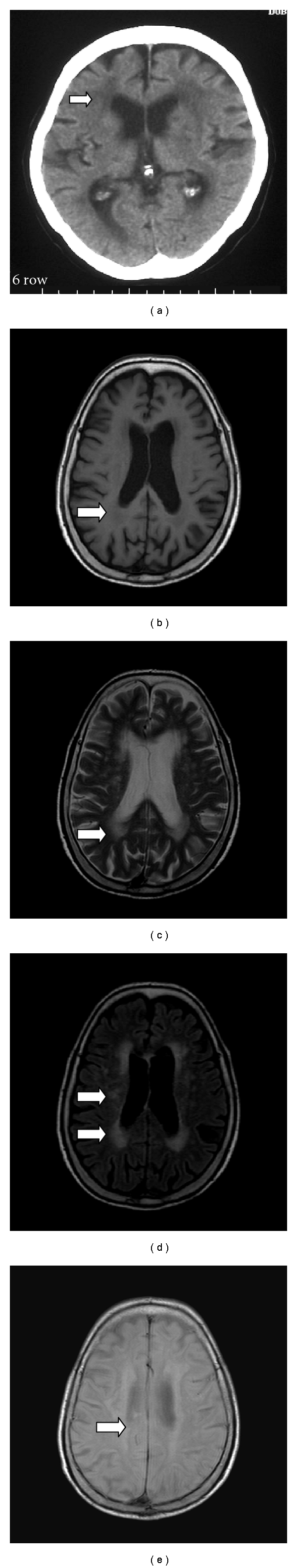
WMC on CT and MRI. (a) Hypodensities on CT; (b) hypointensities on T1-weighted MRI; (c, d, e) hyperintensities on T2-weighted MRI, proton-density, and FLAIR sequences, respectively. (d) The lower arrow shows the PVWMC, and the upper one shows the DWMC.

WMC are ill defined hypodensities on CT. On MRI, which is more sensitive than CT on delineating the lesions, they appear as hypointensities on T1-weighted imaging and hyperintensities on T2-weighted imaging, proton density and fluid-attenuated inversion recovery sequences (FLAIR) (Figure 1). The FLAIR sequence is probably the best to assess the severity of WMC because of clear distinction between ventricles and the PVWMC. Lesions are defined as PVWMC when their largest diameters are adjacent to the ventricles, otherwise they are considered as DWMC [75]. The more recent diffusion tensor imaging (DTI) technique provides information on the integrity of white matter tracts by estimation of the diffusion trace (mean diffusivity) and the directionality-fractional anisotropy (FA). In WMC, mean diffusivity is elevated and FA is reduced [76], which suggests impaired white matter integrity. Measures of DTI are probably more sensitive than WMC volume in detecting cognitive changes over time [77].

As to the assessment of WMC severity, various visual rating scales have been proposed. The visual rating scales are quick and easy to perform on different quality of scans [78]. However, they varied from each other, and data is not quantitative and has ceiling effect. In addition, discrepancies between the various scales may lead to inconsistent findings. Two most popular visual rating scales are Fazekas scale [79] which has been validated histopathologically and Scheltens scale [80] which is detail with good reliability but relatively time-consuming. To unite the visual rating for WMC, the vascular cognitive impairment harmonization standard recommends the age-related WMC (ARWMC) scale [81] as the preferred visual rating scale [82]. This scale can be applied to both MRI and CT with moderate to excellent reliability, and it has been validated against volumetric measurement and cognitive impairment [83]. The operationalized ARWMC scale further gave operational definitions on ARWMC scale and improved interrater reliability on CT [84]. Noteworthy is that visual rating scale for assessing the progression of WMC is lacking. Both the Rotterdam progression scale [85] and Schmidt progression scale [42] were correlated with volumetric measurements. The Rotterdam progression scale had moderate to good reliability (weighted Cohen's = 0.63 (intraobserver), 0.59 (interobserver)), whereas the Schmidt progression scale was less reliable [85, 86].

Nowadays, fully automated techniques and semiautomated segmentation methods become increasingly available. Different from the visual rating scales, volumetric measurement is more accurate and provides continuous data without ceiling effect. However, it is more time-consuming with higher requirement of expertise and excellent quality of MRI, which limits its use for research purpose. Overall, volumetric method is preferred over visual rating scales for longitudinal studies.

## 6. Clinical Importance

### 6.1. Cognitive Impairment and Decline

Large amounts of evidence shows that WMC are associated with cognitive impairment (executive function [2, 87, 88], mental processing speed [89, 90], and global cognition [2, 91]) and long-term cognitive decline in both community and stroke patients [92]. Although some inconsistent results exist, part of the discrepancies stem from different sensitivities of rating scales for WMC, small sample size, and use of different neuropsychological tests [60]. Longitudinal studies have demonstrated that WMC progression parallels cognitive decline [92]. Baseline PVWMC volume was longitudinally associated with reduced mental processing speed [93] and increased the risk of dementia [94, 95]. In some studies, when brain atrophy was added into the predicting models, influence of WMC became insignificant whereas global and/or regional atrophy (i.e., medial temporal lobe atrophy, cortical gray matter (cGM) and hippocampus atrophy) exerted greater influence upon cognitive decline [96–98]. These studies also showed that whole brain or cGM atrophy was related to severity of WMC. It was thus proposed that cognitive decline in patients with WMC was mediated by brain atrophy [96]. The hypothesized mechanisms of how WMC induce cortical atrophy include demyelination of axons leading to cortical-subcortical deafferentation and subsequent secondary cortical neuronal loss, [99, 100] hypoperfusion, and hypometabolism [101, 102], as well as concomitant cortical microinfarct, which its detection is beyond the ability of current neuroimaging techniques [102].A recent study also showed that severe WMC were associated with hippocampus atrophy [103]. Moreover, Smith et al. study in community residents revealed that WMC progression might predict normal to mild cognitive impairment, whereas global atrophy predicted mild cognitive impairment to dementia [104]. With the development of DTI technique, studies indicated that microstructural integrity of both WMC and normal-appearing white matter was associated with cognitive function, regardless of white matter atrophy, WMC volume, and lacunar infarcts [105, 106].

Certain factors need to be considered in the evaluation of the relationship between WMC and cognitive impairment. First, the relationship between WMC and cognitive impairment may not be linear, and a threshold effect was proposed [107]. Second, cognitive impact of WMC may vary with its location. PVWMC may affect cognition more than DWMC. Thus, studies evaluating total WMC may dilute the cognitive influence of region-specific WMC [108]. Third, psychometric tests in studies may not be comprehensive for the assessment of executive function; hence, the impact of WMC may be underestimated [108]. Forth, silent brain infarcts and microbleeds were reported to be associated with cognitive impairment as well [109–113], and brain atrophy (cGM, hippocampus and medial temporal lobe atrophy) may be a confounder between WMC and cognitive impairment, these neuroimaging measures were not assessed in some studies. Last, future studies should utilize DTI in exploring the mechanisms of cognitive decline and as a surrogate marker for disease progression in therapeutic trials.

### 6.2. Gait Disturbance and PD

Both cross-sectional and longitudinal studies have found that WMC were associated with gait disturbance and falls [114–121]. Tasmanian Study of Cognition and Gait study showed that the risk of incident falls was doubled in people with WMC volumes in the highest quintile of its distribution compared with the lowest (adjusted relative risk 2.32, 95% confidence interval: 1.28–4.14) [122]. Regardings the WMC location on gait, Tasmanian Study of Cognition and Gait study found that bilateral frontal and periventricular WMC-affected voxels corresponded to major anterior projection fibers (thalamic radiations, corticofugal motor tracts) and adjacent association fibers (corpus callosum, superior frontooccipital fasciculus, short association fibers) showed the greatest covariance with poorer gait [123]. A DTI study also found white matter integrity in the genu of corpus callosum was an important marker of gait in the elderly [124]. Another two recent DTI studies revealed that in elderly subjects with small vessel disease, widespread disruption of white matter integrity, predominantly in the normal-appearing white matter, was involved in gait disturbances [125, 126]. Iseki et al. study using single-photon emission-computed tomography suggested that abnormalities in the basal ganglia-thalamocortical loops partly explained gait disturbance in WMC [127]. Thus, accumulating evidence suggests that the disruptions in motor network may account for gait disturbance in WMC.

WMC correlate with gait disturbance in community residents, and some of these abnormalities overlap with features of PD. In postmortem study with 700 parkinsonism cases, 27 brains (3.9%) showed WMC and/or lacunes in the basal ganglia, white matter, or brainstem, without significant nigral lesions [128]. Studies also found that WMC contributed to dementia in PD patients [16, 129, 130]. Albeit inconclusive, evidences accumulating that cognitive effects of WMC tend to preferentially affect executive functions and may reflect frontal lobe WMC [129].

### 6.3. Urinary Incontinence

Studies had shown that WMC were associated with urgency urinary incontinence [131–135]. A community study found that among 100 residents, 64% of them had urinary incontinence. The presence of WMC in right inferior frontal regions and selected WM tracts predicted incontinence, incontinence severity, and degree of bother. The study confirmed a critical role for the cingulum in bladder control and suggested potential involvement of anterior corona radiata and superior frontooccipital fasciculus [132]. A study in old women indicated that the presence of WMC in specific pathways (anterior thalamic radiation and superior longitudinal fasciculus) might affect continence control [136].

### 6.4. Depression

Lines of evidence suggested that WMC are associated with late life depression [45, 137–144]. In poststroke patients, severe deep WMC predicted poststroke depression [145]. And concurrent atrophy of left inferior frontal gyrus was associated with depressive symptoms in poststroke patients with severe WMC [146]. Vascular depression hypothesis proposed that WMC causes depression by disrupting fiber tracts within frontostriatal circuits [142]. Because of their involvement in the regulation of mood, disruption of frontostriatal circuits might lead to a disconnection syndrome that corresponded to the clinical and neuropsychological profile of depression [142]. A DTI study also found that frontolimbic neural pathways might contribute to the pathophysiology of depression [147].

### 6.5. Stroke and Death

The WMC increased the risk for stroke [8, 95, 148, 149] and death [149–151]. A recent meta-analysis revealed that stroke yielded a significant association of WMC with incident stroke (HR 3.5 (2.5–4.9), *P* < 0.001) and increased risk of death (HR 2.0 (1.6–2.7), *P* < 0.001) [92]. In patients treated with thrombolysis for acute stroke, the rate of symptomatic intracerebral hemorrhage increased by 10% in patients with severe WMC and multiple lacunes [152]. WMC were found to be independent determinants for intracerebral hemorrhage after controlling for age and other risk factors [152, 153]. 

WMC were not benign but predict poor clinical and functional outcomes. Some studies also indicated that they, especially DWMC, were associated with migraine [154, 155]. And brain stem WMC were associated with dizziness [156]. More studies are in need to explore the clinical significance of WMC.

## 7. Chemical Biomarkers

Homocysteine is a dietary sulphur-containing amino acid derived as an intermediate during the metabolism of methionine [157]. Nutritional deficiencies in the vitamin cofactors (folate, vitamin B_12_, and vitamin B_6_) required for homocysteine metabolism may promote hyperhomocyst(e)inemia [158]. Perini et al. study found that the hyperhomocysteinemia in acute stoke stage was associated with higher risk of small artery disease subtype of stroke [159]. Many studies had indicated that hyperhomocysteinemia was an independent predictor for WMC independent of smoking, hypertension, or age [160–172].

Inflammatory biomarkers as intercellular adhesion molecule-1 (ICAM-1) [173, 174], and high sensitive C-reactive protein (Hs CRP) [175–177] were also reported to be associated with WMC load. However, these studies were cross-sectional so that causal relationship cannot be determined. Longitudinal studies are needed to study the relationship between progression of inflammatory factors with progression of WMC.

## 8. Treatment of WMC

The WMC are predictors for poor clinical outcomes and important substrates for vascular dementia. The European Task Force on age-related WMC recommended that clinical trials on cerebral small vessel disease should target those with severe WMC and use its progression as surrogate marker in clinical trials [49]. Albeit WMC are clinically important, very few clinical studies had been conducted so far to evaluate treatments for WMC. In this part, studies on treatments for WMC- and WMC- related vascular dementia are reviewed.

### 8.1. BP Lowing Therapy

The Epidemiology of Vascular Ageing MRI study has shown a positive linear relationship between BP and severity of WMC [28]. Dufouil et al. study retrospectively found subjects receiving regular treatment of hypertension had less severe PVWMC than those receiving no or irregular treatment of hypertension. Due to its cross-sectional design, the treatment effect of hypertension on WMC progression cannot be examined in this study. The Perindopril Protection against Recurrent Stroke Study (PROGRESS) MRI substudy [178] was a longitudinal randomized placebo-controlled trail investigating the BP lowing therapy using perindopril or perindopril plus indapamide on WMC progression. 192 participants were followed up for 36 months, the mean total volume of new WMC was significantly lower in the active treatment group compared with the placebo group, and the difference was greatest for patients with severe WMC at entry [178]. 

An open-label study [179] and a post hoc analysis of a randomized trial [180] showed some beneficial effects of nimodipine in patients with subcortical vascular dementia. A recent randomized placebo-controlled trial also found that the calcium antagonist nimodipine could slow down global cognitive decline in patients with small vessel disease related vascular dementia [181]. A recent review in vascular cognitive impairment indicated that nicardipine has been investigated in more than 6000 patients, with improvement of cognitive deterioration in more than 60% of patients treated [182]. The antihypertensive activity of nicardipine and its safety and effectiveness in cognitive domain suggested reconsidering this drug in the treatment of cognitive impairment of vascular origin and for reducing the risk of recurrent stroke in patients at high risk of it [182]. Further randomized double-blind placebo-controlled trail is needed to explore the efficacy and safety of nimodipine and nicardipine upon WMC progression. 

Although most studies paid attention to lower BP in hypertension, noted that low BP was a risk factor for WMC as well [60]. Another study found that nocturnal BP dipping was associated with WMC [183]. Thus, a randomized controlled study dedicated to examine the effects of BP lowering therapy upon progression of WMC is needed.

### 8.2. Statins

Statins have long been demonstrated to reduce cardiovascular events and ischemic stroke among patients with coronary heart disease [184]. Whether statins affect progression of WMC is still controversial. The PROSPER (Prospective Study of Pravastatin in Elderly at Risk) study examined the effect of pravastatin 40 mg daily on the progression of WMC in 270 placebo-treated subjects and 265 active subjects within a period of 33 months. The study failed to demonstrate an overall beneficial effect of statins upon WMC progression. However, data on proportions of subjects having different WMC severity are lacking, and stratified analysis based on WMC severity was not performed in the study. In the Cardiovascular Health Study, 3334 community participants were followedup over an average observational period of 7 years [185]. Patients treated with statins were observed to have slightly less cognitive decline than untreated subjects. This significant cognitive benefit was associated with reduced progression in cerebral infarcts among the treated subjects, whereas progression of WMC was not statistically different between these two groups. Although the findings may suggest that statins exert cognitive benefits independent of WMC progression, the visual rating scale used in that study was unlikely to be sensitive in detecting WMC progression [38]. The ROCAS (Regression of Cerebral Artery Stenosis) study evaluated simvastatin on WMC progression in patients with asymptomatic middle cerebral artery stenosis [38]. Two hundred and eight randomized subjects were assigned to either placebo (*n* = 102) or simvastatin 20 mg daily (*n* = 106) for 2 years. Simvastatin group did not slow the progression of WMC volume compared with the placebo group, but in those with severe WMC at baseline, the median volume increase in the simvastatin group (1.9 cm^3^) was less compared with that in the placebo group (3.0 cm^3^; *P* = 0.047). However, in this study, treatment probably prevented WMC progression among those with severe WMC at baseline was based only on subgroup analysis upon a small subset of subjects. Furthermore, the subjects of this study belonged to a high-risk group in that all our subjects had concurrent MCA stenosis. Hence, the findings may not be applicable to patients with less vascular burden or to those with WMC but without concurrent MCA stenosis.

Furthermore, a recent cross-sectional study showed that low cholesterol had more severe WMC in acute stroke patients [10]. Other studies found that low cholesterol was associated with intracerebral hemorrhage and high mortality in these patients [186, 187]. Hence, effects of stains and cholesterol control upon WMC progression are still uncertain, and randomized clinical trials are needed to address this issue.

### 8.3. Acetylcholinesterase Inhibitors and *N*-Methysl-D-Aspartate (NMDA) Receptor Antagonists

Acetylcholinesterase inhibitors (donepezil, galantamine, and rivastigmine) and *N*-methyl-D-aspartate (NMDA) receptor antagonists (memantine) have been approved for treatment of AD. Kavirajan and Schneider reviewed three donepezil, two galantamine, one rivastigmine, and two memantine placebo-controlled, randomized, double blinded trials, it showed that cognitive effects on the AD assessment scale-cognitive subscale (ADAS-cog) were significant for all drugs, and post hoc analyses of donepezil tails suggested greater improvement in patients with cortical and territorial lesions compared with those with predominantly subcortical small vessel disease related lesions [188]. By contrast, cognitive benefits in the memantine trials appeared to be more pronounced for patients with small vessel disease than for those with large vessel disease and such benefits derived largely from worsening in patients in the placebo-treated groups who predominantly had small vessel disease [188, 189]. With regards to safety profile, use of cholinesterase inhibitor significantly increased the odds of having adverse events (e.g., anorexia, nausea, vomiting, and diarrhea), while memantine was found to be well tolerable and safe. Overall, the data is insufficient to support the widespread use of acetylcholinesterase inhibitors (donepezil, galantamine, and rivastigmine) and memantine in patients with vascular dementia [188]. Yet, given the potential benefit of memantine upon subcortical vascular dementia based on post hoc analysis and its favorable safety profile, conducting a randomized study evaluating its efficacy in subcortical vascular dementia may be worthwhile. 

Cerebral autosomal dominant arteriopathy with subcortical infarcts and leucoencephalopathy (CADASIL) is a genetic form of subcortical vascular dementia. A recent multicentre, 18-week, placebo-controlled, double-blind, randomized parallel-group trial using donepezil in 168 CADASIL patients revealed that donepezil had no effect on the vascular ADAS-cog score in CADASIL patients with cognitive impairment. Improvements were noted only on several measures of executive function, which might possibly suggest that cholinergic pathways were involved in the executive function [190]. Overall, findings of this study are similar to that of previous studies using acetylcholinesterase inhibitors in that acetylcholinesterase inhibitors might only induce subtle cognitive benefits among patients with vascular dementia.

Another more recent randomized, international, multicenter, 24-week trial in 974 probable or possible vascular dementia patients who received donepezil 5 mg/d or placebo found that donepezil improved the vascular ADAS-cog score but not global function [191]. However, subgroup analysis on the effects of donepezil upon subcortical type of vascular dementia was not performed in this study.

### 8.4. Homocysteine Lowering Therapy

Lines of evidence have shown that hyperhomocysteinemia was associated with WMC through endothelial dysfunction [160–172]. Whether homocysteine lowering therapy by means of multivitamins retards the progression of WMC or not is uncertain. A randomized double-blind, parallel, placebo-controlled trial on homocysteine lowering therapy is the VITAmins TO Prevent Stroke (VITATOPS) study.8164 patients with recent stroke or transient ischaemic attack (within the past 7 months) received one tablet daily of placebo (*n* = 4089) or B vitamins (2 mg folic acid, 25 mg vitamin B_6_, and 0.5 mg vitamin B_12_,  *n* = 4075) with a median followed-up duration of 3.4 years. Although vitamin treatment was not significantly more effective than placebo in reducing the incidence of the composite primary endpoint of stroke, myocardial infarction, or vascular death, in the subgroup analyses, homocysteine lowering might have preferential benefit in small vessel disease patients (risk ratio 0.80 (95% CI : 0.67–0.96)) [192]. The VITATOPS MRI substudy is currently underway to evaluate whether vitamins can slow WMC progression and/or cognitive decline.

## 9. Conclusion

WMC are common in elderly, and they are not benign. More extensive WMC are associated with a host of poor clinical outcomes. Although WMC have been shown to be associated with small vessel disease, age, and other vascular risk factors, the exact mechanisms explaining such association are still uncertain. To date, data on the effectiveness of various treatments (e.g., BP lowering, statins) in preventing WMC progression were derived mainly from subgroup analyses. Randomized studies dedicated in evaluating treatments for preventing WMC progression and its clinical correlates are thus urgently needed. Although some studies have suggested the efficacy of nimodipine, nicardipine, and memantine in subcortical vascular dementia, further randomized controlled studies are needed to clarify their effectiveness and safety.
